# Vaccina Developed after Fourteen Years Incubation of the Virus

**Published:** 1879-07-20

**Authors:** Lindsay Johnson

**Affiliations:** Cartersville, Ga.


### *VACCINA DEVELOPED AFTER FOURTEEN YEARS INCUBATION OF THE VIRUS.*

BY LINDSAY JOHNSON, M. D., CARTERSVILLE, GA.

Some time in 1877, I was called to see J. A. Howard, Ordinary of Bartow county, Ga., to examine a sore on his right arm, which was giving consideraole annoyance on account of the peculiar burning pain resulting therefrom. Being impressed with the significant appearance of the vesicle—for such it was—I at once asked him when he had been vaccinated? He replied not since 1863, while a soldier in the Confederate service.

Becoming deeply interested in the case, I insisted on his giving a history of the vaccination and of his general condition since. The following facts were elicited :

Was vaccinated about the middle of March, 1863, while stationed at Savannah, Ga., by Dr. A. P. Brown, the regimental surgeon. The virus used was said to be perfectly pure, being furnished by the State government authorities. No result followed the vaccination, save the appearance of one or two *very small* red pimples, which remained for a day or so, and dried up entirely. The slight redness around the pimples fading away by the third day after vaccination.

For eight or ten years previous to the appearance of the present vesicle, patient has been afflicted at short intervals with the most dreadful furuncles, etc., invariably accompanied by considerable febrile excitement, with nausea and vomiting, followed by extreme nervous prostration; again, in the absence of inflammation, circumscribed or otherwise, he would experience frequent attacks of rigors, followed by fever of variable degrees of intensity which would leave him very much depressed in spirit and muscular ability. After having gone through one of these attacks, which would last, according to the phase of symptoms assumed, his general health would be good. Nervous and muscular force considerably above the average; would exhibit a plentiful supply of vigor, and indeed would seem altogether free from care and annoyance of any kind; then without the slightest admonition, he would have a visitation of his old and dreaded troubles.

Right here I will state in justice to the patient and in behalf of his morals, that he has never been the victim of any kind of venereal disease; in fact his has been a character worthy of emulation in strict habits of virtue.

I have thus endeavored to give the history, as elicited from the patient, of a case which, I think, may be regarded as one of great peculiarity, since after an extensive and thorough search I can find nothing that approaches similarity.

My first examination was made on the fifth day after the appearance of a red pimple, as described by patient. Each day I made a close inspection of the vesicle in its progress, and as it continued to grow larger, becoming thoroughly satisfied that my suspicions were correctly founded. About the eighth day the vesicle was surrounded by a bright red areola; on the eleventh day this bright red ring began to fade, the vesicle drying and becoming umbilicated, exhibiting a thick, flat scab, depressed in the center, and showing plainly all the marks of a typical case, confirming the fact of it being the result of vaccine virus introduced into the arm at Savannah, Ga., fourteen years ago, the patient *positively* averring that he has not been vaccinated since; and the cicatrix to be seen to-day would thoroughly convince the most skeptical, and in this connection I will say that the cicatrix was minutely criticized on the day this article was written, by two eminent physicians of this county, Drs. Stephens and Sims, both pronouncing it to be genuine, without a moment's hesitation.

I shall not offer an hypothesis in explanation of the latent action of the vaccine virus in this particular case; certain it is that vaccine virus causes a zymotic action to take place in the circulatory system, which system furnishes a material that combines with the *materies morbi* of the virus, thus giving rise to the vaccine disease, by which means this material in the body is exhausted, thereby lessening the susceptibility to variola to such an extent as to furnish almost complete immunity for life.

In conclusion, I will add that since this attack of vaccine disease which was accompanied by greater febrile excitement, restlessness, etc., than usually attends the disease, the patient has enjoyed perfect health, as his looks now betokens, and has never felt the slightest indication of a return of his former troubles.

The desire to obtain the views of the profession in a case I look upon to be without a precedent, has led me to give, in my plain style, this history, and I earnestly and deferentially await the expression of those whose experience and ability entitle them to go before.

